# A practical method for reducing the interference due to lipaemia in coagulation tests

**DOI:** 10.1111/ijlh.13129

**Published:** 2019-11-26

**Authors:** Chris Gardiner, Philip Lane, Hitesh Tailor, Ian J. Mackie

**Affiliations:** ^1^ Haemostasis Research Unit University College London London UK; ^2^ Haematology Evaluations Unit HSL (Analytics) LLP London UK

**Keywords:** coagulation, laboratory automation, laboratory practice

## Abstract

**Introduction:**

Plasma samples with gross lipaemia present a challenge for coagulation laboratories using optical analysers. High‐speed centrifugation may be used to remove excess lipids but it has not established whether this affects haemostasis tests. The aims were to determine whether the removal of lipid by centrifugation affects PT, APTT, fibrinogen, D‐dimer and von Willebrand factor activity measurements.

**Methods:**

Twenty‐six lipaemic samples (median [range]): triglyceride 4.6 mmol/L [0.5‐17.0]; cholesterol: 4.06 mmol/L [2.20‐9.41] and 20 plasmas spiked with Intralipid 20 or lipid isolated from patient plasmas (median triglyceride of 11.95 mmol/L [5.0‐17.0] and cholesterol 4.33 [3.22‐7.06]), were tested before and after the removal of the lipid layer by centrifugation (10000 *g* for 10 minutes). Tests were performed using the CS‐5100 (Sysmex) coagulation analyser.

**Results:**

Thirteen, 9, 3 and 1 of the lipaemic or spiked samples failed to give PT, APTT, fibrinogen and D‐dimer results, respectively. Centrifugation significantly reduced triglyceride (median 2.7, [0‐6.1 mmol/L]) and cholesterol (median 0.52 [0‐3.5]), allowing clot detection in all tests. There were no statistically significant differences in fibrinogen, D‐dimer or VWF levels in samples before and after lipid removal. A small but clinically insignificant change in PT and APTT was observed after lipid removal.

**Conclusion:**

High‐speed centrifugation reduces lipaemia sufficiently to allow testing on an optical coagulation analyser without introducing clinically significant differences PT, APTT, fibrinogen, D‐dimer or VWF activity values.

## 1 INTRODUCTION

Lipaemic plasma samples are commonly encountered in hospital diagnostic laboratories. Excessive lipaemia can increase turbidity in plasma samples due to an excess of large lipoprotein particles, especially chylomicrons.^1^ Consequently, plasma samples with lipaemia present a challenge for clinical laboratories using optical analysers since light scatter and absorption may be increased resulting in reduced light transmission.^2^ This may result in instrument error flags, requiring visual inspection of coagulation curves or, in the presence of gross lipaemia, instrument vote‐outs (ie no result is generated). Lipaemia may be the result of postprandial blood lipid changes or following lipid infusions in critically ill patients. In these cases, a repeat sample taken from a fasting patient or prior to lipid infusion may eliminate the problem,^3^ but this leads to delay in obtaining results, which could delay diagnosis or treatment. However, if the lipaemia is due to a medical/metabolic condition (eg familial hypertriglyceridaemia, diabetes mellitus, renal and liver disease), repeat testing will not eliminate the problem. In these cases, the only alternative is retesting with a mechanical coagulometer or manual tube tilt‐tube method in a water bath. However, many clinical laboratories do not have the facilities to do this.

Several methods have been proposed to reduce optical interference due to lipaemia, including ultracentrifugation (>60 000 *g*); high‐speed centrifugation (8000‐20 000 *g*); and lipid extraction using organic solvents or lipid‐clearing agents. The Clinical and Laboratory Standards Institute (CLSI)^4^ states that “ultracentrifugation has been suggested in some circumstances; however, although there is anecdotal evidence to suggest that this is widely used in haemostasis laboratories, there are no published studies.” Ultracentrifugation is unavailable to most clinical laboratories, and furthermore, it may result in the loss of high molecular weight analytes such as fibrinogen, the FVIII/von Willebrand factor complex and D‐dimer.^2^ Dimeski et al^5^ compared the effect of high‐speed centrifugation of lipaemic serum samples at 20 000 *g *and ultracentrifugation on biochemistry results. Although ultracentrifugation removed much more triglyceride and cholesterol than high‐speed centrifugation, the latter removed enough of the large lipoproteins to allow testing without optical interference. Furthermore, high‐speed centrifugation produced results closer to those obtained in the original uncentrifuged plasma.

We propose a simple high‐speed centrifugation method to reduce the level of lipaemia to a level which allows testing on an optical analyser without substantially altering the test results in a range of haemostasis parameters.

## 2 METHODS

### 2.1 Samples

Blood samples were collected into 0.109 M sodium citrate (Vacutainer^®^, Becton Dickinson), and plasma was prepared by centrifugation at 2000 *g* for 15 minutes.

Twenty‐six plasma samples (hereafter referred to as “lipaemic samples”) with a median triglyceride concentration of 4.6 mmol/L (range 0.5‐12.5) and median cholesterol concentration of 4.06 mmol/L (range 2.20‐9.41) were obtained from residual, anonymized samples collected after all diagnostic testing had been completed, in compliance with local ethical committee rules and the Human Tissue Act.

Twenty plasmas from normal volunteers were spiked with exogenous lipoproteins (hereafter referred to as “spiked samples”): ten with 5 µL/mL Intralipid 20 (Sigma‐Aldrich) and ten with lipid isolated from lipaemic patient plasmas, to give a median triglyceride concentration of 11.95 mmol/L (range 5.0‐17.0) and median cholesterol concentration of 4.33 (3.22‐7.06). Informed consent was obtained from normal donors (approved by the UCL Research Ethics Committee: Project ID Number: 7029/001).

### 2.2 Reagents

Tests were performed using the following reagents (All Siemens Healthcare) on a Sysmex CS‐5100 coagulation analyser (Sysmex Corp):

Prothrombin time (PT)—Dade^®^ Innovin^®^ PT Reagent.

Activated partial thromboplastin time (APTT)—Dade^®^ Actin^®^ FS Activated PTT Reagent.

Clauss Fibrinogen (Fib)—Dade^®^ Thrombin Reagent and Owren's Veronal Buffer.

D‐dimer—INNOVANCE^®^ D‑Dimer.

Von Willebrand activity (VWF Ac)—INNOVANCE^®^ VWF Ac.

Von Willebrand ristocetin cofactor (VWF:RCo)—BC von Willebrand Reagent.

The manufacturer states on their application sheets that there is no interference in the above tests up to these stated triglyceride levels (mmol/L): PT 2.28, APTT 2.29, Fib 3.26, D‐dimer 3.39, VWF Ac 10.04 and VWF:RCo 6.77. No interference values for cholesterol are stated in their literature.

Cholesterol and triglycerides were measured using Chol2 and triglyceride reagents using a cobas C111 analyser (Roche Diagnostics).

### 2.3 Lipid reduction method

Plasma was aliquoted into a 1.8‐mL conical microcentrifuge tube and centrifuged at room temperature at 10 000 *g* for 10 minutes in an Eppendorf^®^ Microcentrifuge 5415 (Thermo Fisher Scientific). The plasma was then carefully transferred into a clean tube using a plastic pipette, leaving the lipid fraction adhering to the microcentrifuge tube. For spiking experiments, the lipid fractions were pooled and washed in sterile isotonic saline, to remove residual plasma. Due to a limited reportable range and sample volume constraints, VWF:RCo testing was only performed on 12 spiked samples.

### 2.4 Haemostasis testing

An aliquot of each lipaemic sample and the corresponding lipid depleted plasma were tested in the same run. In the spiking experiments, an aliquot of the unspiked plasma, an aliquot of spiked plasma and an aliquot of lipid depleted plasma were tested in the same run.

### 2.5 Statistical analysis

Statistical analysis was performed using GraphPad Prism v8. Nonparametric tests were used throughout.

## 3 RESULTS

**Figure 1:**
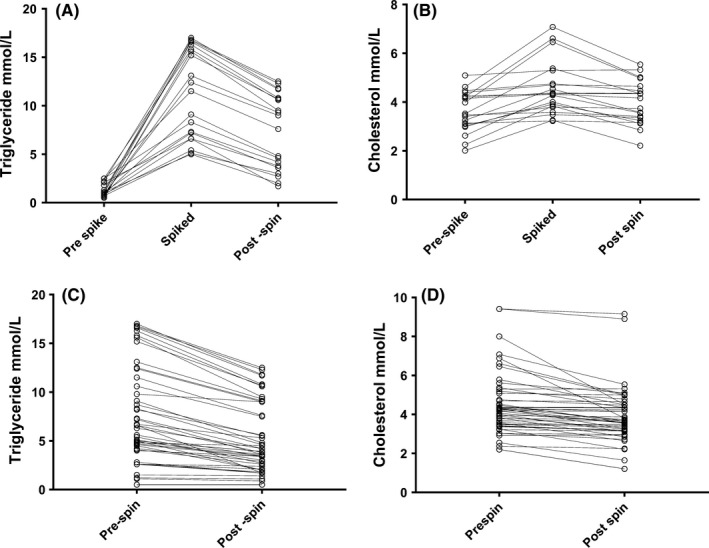
The effect of lipid depletion on plasma triglyceride and cholesterol concentration. A, Triglyceride in native plasma, postspiking and postlipid depletion. B, Cholesterol in native plasma, postspiking and postlipid depletion. C, Triglyceride in lipaemic samples pre‐ and postlipid depletion. D, Cholesterol in lipaemic samples pre‐ and postlipid depletion

The removal of the lipid fraction from lipaemia samples significantly reduced the concentration of triglyceride (median reduction 1.1, [0‐3.4 mmol/L] *P* < .0001) and cholesterol (median 0.29 mmol/L [0‐3.5] *P* < .0001) (Figure 1A‐B). When the lipid fraction was removed from the spiked samples, a similar reduction in triglyceride (median reduction 4.1 mmol/L [range 0.0‐3.4] *P* < .0001) and cholesterol (median reduction 0.48 mmol/L [range 0.0‐1.59) concentration was observed. However, the triglyceride and cholesterol concentrations postlipid removal remained significantly higher than the prespike sample (Figure 1C‐D).

Prothrombin time results were not produced on four lipaemic samples and nine spiked samples due to low light transmission, that is turbidity. APTT vote‐outs were seen in two lipaemic and seven spiked samples, while one lipaemic sample and two spiked plasmas did not produce a fibrinogen result and one lipaemic sample failed to produce a D‐dimer result. The median triglyceride concentration in samples failing to produce results for one or more tests was 10.6 mmol/L (range 5.0‐17.0), and median cholesterol for these samples was 5.30 mmol/L (range 2.20‐8.00). All plasmas gave results for all assays after high‐speed centrifugation.

**Figure 2:**
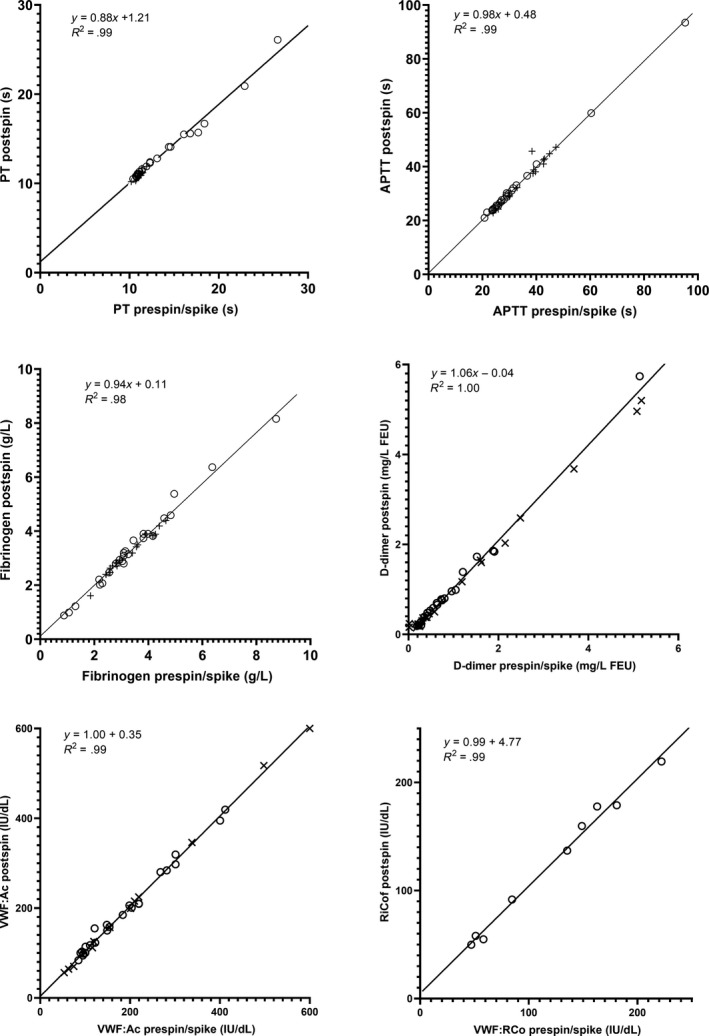
The effect of lipid depletion on haemostasis assays (lipaemic samples o, spiked samples x)

For the statistical analysis (Table 1), the results of prespin lipaemic samples and prespiked plasmas were combined into a single group and compared with the results of postlipid depletion plasmas. Results for lipaemic samples which did not produce results before centrifugation were excluded from the analysis for the test that was affected. No significant differences in fibrinogen, D‐dimer, VWF Ac or VWF:RCo were observed. PT and APTT results were slightly shorter but, although the differences were statistically significant, they were too small to be of clinical significance (Table 1). Excellent correlation was observed for all tests (Figure 2).

**Table 1:** Median results for plasmas prior to and after lipid reduction by high‐speed centrifugation. Only those results with both pre‐ and postlipid reduction values were included in the analysis

Test	Samples with results	Prespin/spike	Postspin	Wilcoxon signed rank test
PT (s)	42	11.35	11.2	*P* = .002
APTT (s)	44	33.02	32.17	*P* = .02
Fibrinogen (g/L)	45	3.42	3.42	NS
D‐dimer mg/mL FEU	45	0.435	0.46	NS
VWF Ac (IU/dL)	46	222.9	224	NS
VWF:RCo (IU/dL)	9	135.3	151.5	NS
Triglyceride (mmol/L)	46	6.6	3.9	*P* < .0001
Cholesterol (mmolL)	46	4.18	3.66	*P* < .0001

## 4 DISCUSSION

Excessive lipaemia can present a challenge to diagnostic haemostasis laboratories using photo‐optical coagulometers due to excessive turbidity. Current options available to the laboratory are retesting with a mechanical clot detection method or the removal of excess lipoproteins by ultracentrifugation. However, there are no published data to support the use of ultracentrifugation for haemostasis and it is not available to most diagnostic laboratories. The only study to use an organic solvent (n‐hexane) for haemostasis samples found significant changes in both PT and APTT.^6^

We have investigated the use of high‐speed centrifugation to reduce turbidity in lipaemic and spiked samples. The spiking experiments were deemed necessary in order to study the effect of lipid reduction in samples with levels of lipaemia which typically prevent photo‐optical coagulometers producing results for all parameters. The levels of cholesterol and triglyceride achieved in our spiking experiments were in the range of those observed in lipaemic clinical samples received by diagnostic haemostasis laboratories. The majority of the triglyceride levels were above those for which the manufacturer said there should be no interference in PT, APTT, fibrinogen and D‐dimer measurements. However, in diagnostic laboratories, triglyceride levels are rarely available, and operators must rely on visual inspection of the sample, analyser HIL flags, or react when the analyser fails to produce a result.

The triglyceride levels in many of the samples would not have been expected to influence VWF levels, based on the manufacturer interference values. However, it was useful to include VWF measurement as well as fibrinogen and D‐dimer, to demonstrate that high‐speed centrifugation did not remove these large, multidomain proteins.

High‐speed centrifugation at 10 000 *g* reduced turbidity sufficiently to allow a range of commonly requested haemostasis tests in all 46 lipaemic and spiked samples, including those that did not produce results before centrifugation unspun. No clinically significant differences in the 6 parameters tested were observed after lipid depletion. It was notable that there were no statistically significant differences in high molecular weight analytes (fibrinogen, D‐dimer, VWF Ac and VWF:RCo) as the result of high‐speed centrifugation. Correlation between haemostasis test results before and after lipid reduction was excellent in all six parameters. Although the reduction in triglyceride and cholesterol was modest, the reduction in turbidity, as assessed by visual inspection and elimination of instrument vote‐outs, was manifested. This would suggest that the largest lipoprotein particles were preferentially removed by high‐speed centrifugation as previously reported.^5^

Our data show that if a result is obtained by the coagulometer, then that result does not change after reduction of lipid; thus, only samples that do not yield a result require high‐speed centrifugation. The limitations of our study were that only one haemostasis analyser with a single set of reagents was studied. The technique described will therefore require validation for other analyser types and reagents. We believe that this simple method for reducing optical interference due to lipaemia may be suitable for most diagnostic laboratories.

## CONFLICT OF INTEREST

The Haemostasis Research Unit has received funding in the form of unrestricted educational grants from Sysmex UK. However, Sysmex UK had no part in the design of the study or preparation of this manuscript.
